# Mesenteric lipoma revealed by chronic abdominal pain: A rare case report

**DOI:** 10.1002/ccr3.5779

**Published:** 2022-04-20

**Authors:** Kais Fourati, Abderrahmen Masmoudi, Amine Zouari, Rami zouari, Jawhar Bradai, Hentati Najmeddine, Salah Boujelbene

**Affiliations:** ^1^ 518993 General surgery Department Habib Bourguiba Hospital Sfax Tunisia; ^2^ 518993 Radiology Department Habib Bourguiba Hospital Sfax Tunisia

**Keywords:** abdominal pain, mesenteric lipoma, surgery

## Abstract

Mesenteric lipoma is a rare entity. It can be asymptomatic or revealed by unspecific clinical symptoms. Complete resection of the lipoma is often proposed to prevent complications. We report a case of mesenteric lipoma revealed by chronic abdominal pain, and we performed a literature review regarding this rare condition.

## Graphical abstract

Mesenteric localization of lipoma is very rare. It may be suspected in patients presenting with chronic abdominal pain. Complete resection of the tumor is indicated for symptomatic or complicated mesenteric lipoma.

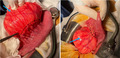

## 1 INTRODUCTION

Lipoma is a rare benign lesion of mature adipose tissue. It commonly occurs in the cephalic part of the body.^1^, ^2^ The localization in the mesentery is extremely rare.^1^ Few cases of mesenteric lipomas have been reported in the literature.^2^ Although mesenteric lipoma is usually asymptomatic, it can be revealed by unspecific clinical symptoms depending on the lipoma size, localization, and the rapidity of its growth.^1^ We report a case of mesenteric lipoma revealed by chronic abdominal pain with a literature review regarding this rare condition.

## 2 CASE REPORT

**FIGURE 1:**
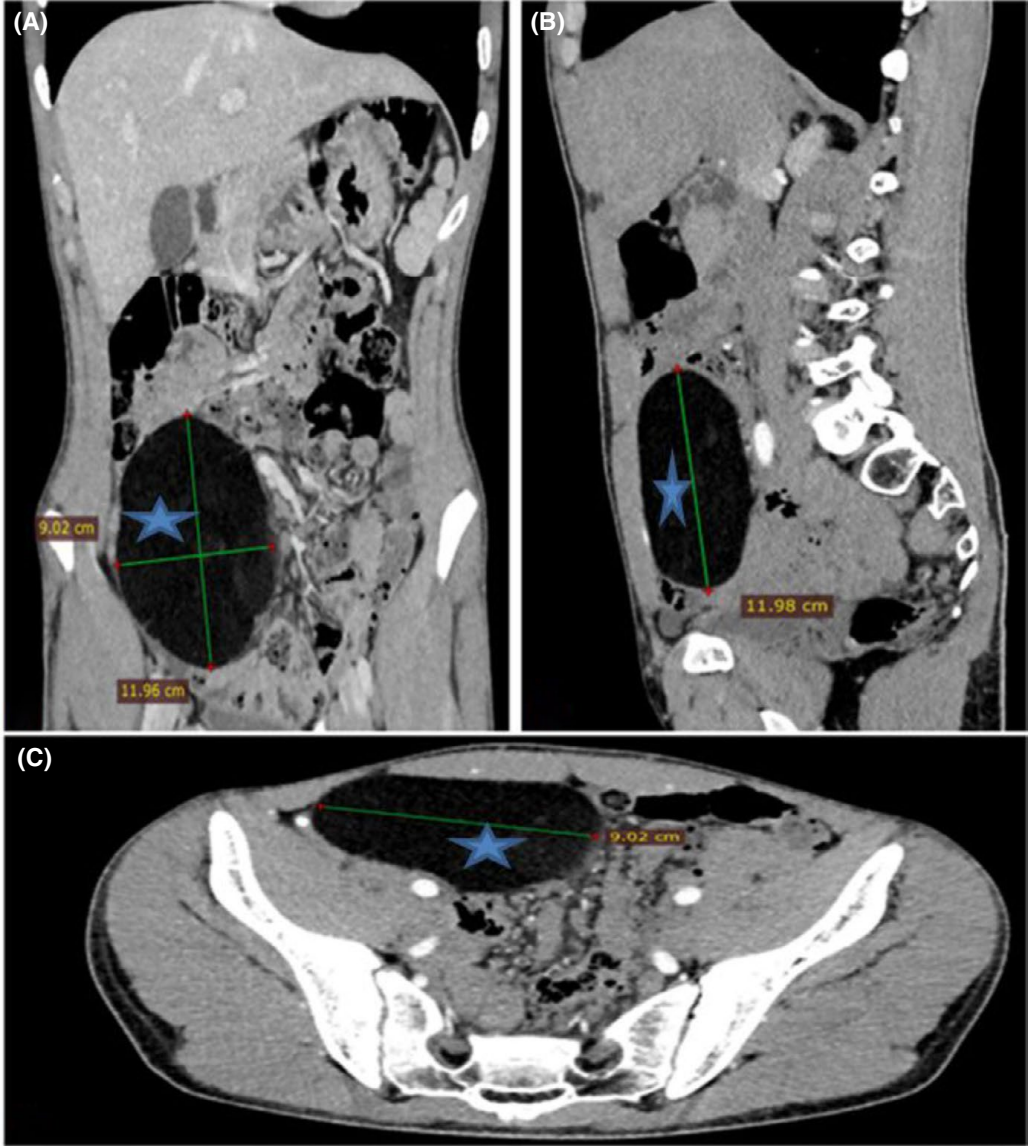
Abdominal CT scan showing mesenteric lipoma localized in the right iliac fossa measuring 12 × 9 cm (blue star)

A 22‐year‐old man with no medical history presented to the hospital with abdominal pain located in the right iliac fossa. The pain began 2 years ago with aparoxysmal evolution, but it has worsened over the last 6 months. The patient also complained of chronic abdominal discomfort. There were no signs of bowel obstruction. Physical examination found a tenderness of the right iliac fossa without a palpable mass. Biology tests were normal including C‐reactive protein. We performed an abdominal ultrasound, which showed a well‐defined, homogenous mass of the right iliac fossa of 10 × 8 cm. The abdominal computed tomography (CT) scan showed a well‐defined mass in the mesentery measuring 12 × 9 cm with fat density surrounded by a thin capsule without any septum or calcification. (Figure 1). This mass did not enhance after injection of contrast product. There was neither lymphadenopathy nor invasion of adjacent organs. The CT scan images concluded to an uncomplicated mesenteric lipoma. Surgery was decided.

**FIGURE 2:**
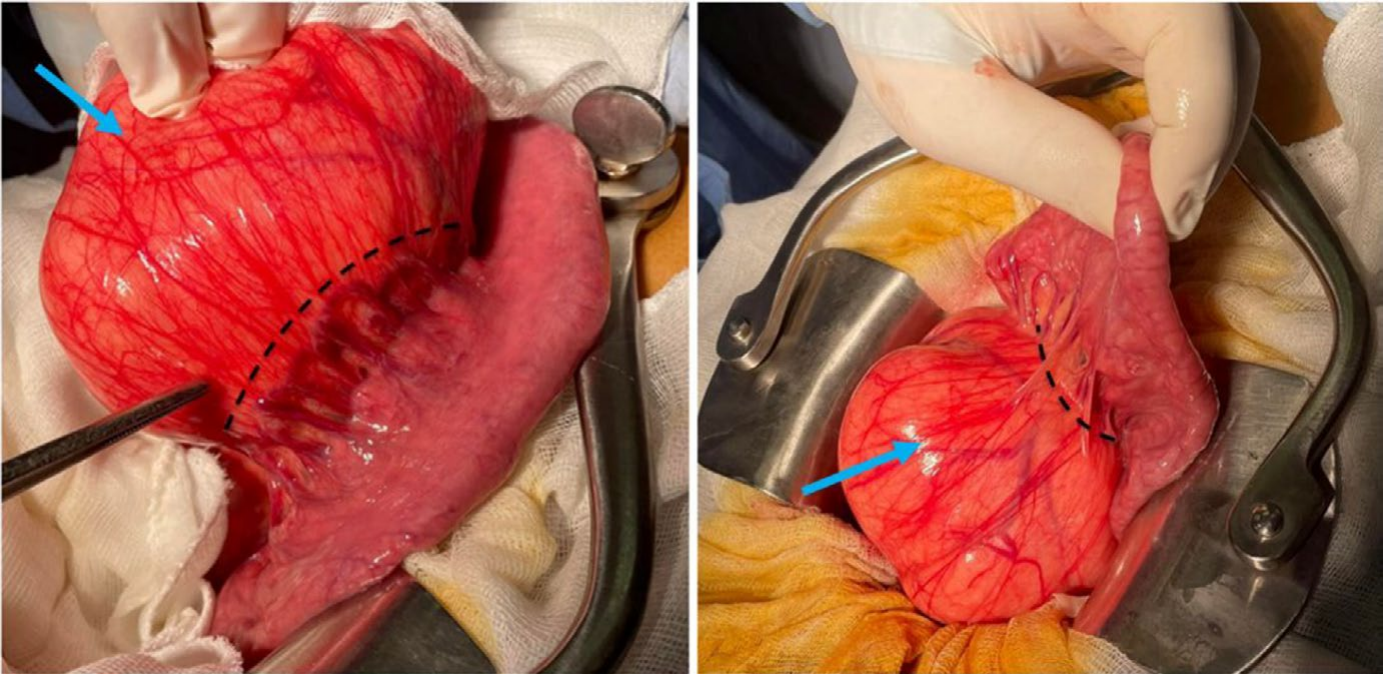
Intraoperative imaging: Dashed lines show the plan of division between the lipoma (blue arrow) and the mesentere

The exploration of the abdomen by midline laparotomy revealed an encapsulated, mobile and yellowish mass originating from the ileal mesentery with no adhesion to other organs (Figure 2).

**FIGURE 3:**
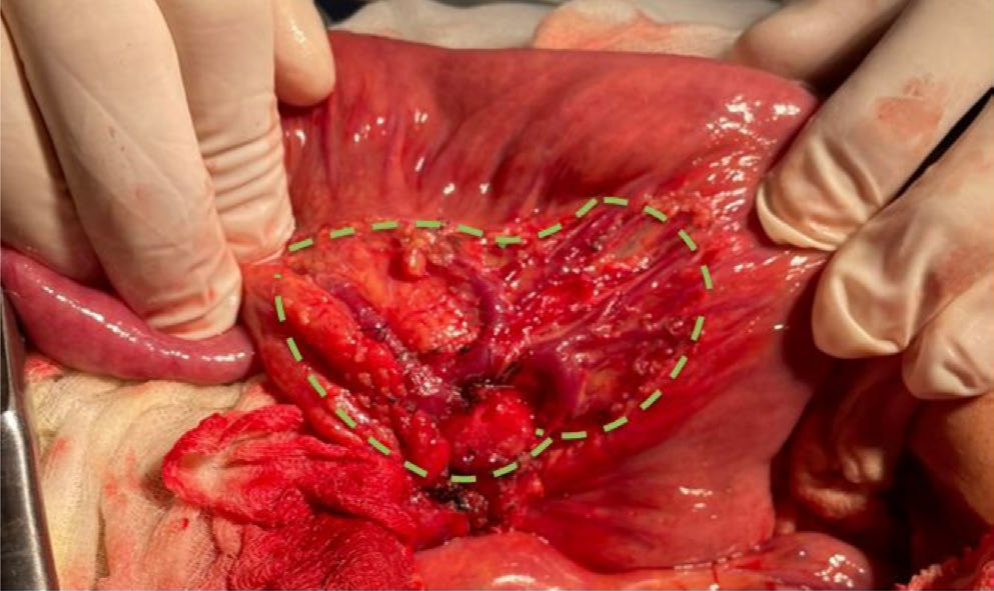
Intraoperative imaging showing the mesenteric bed kept intact (dashed lines)

**FIGURE 4:**
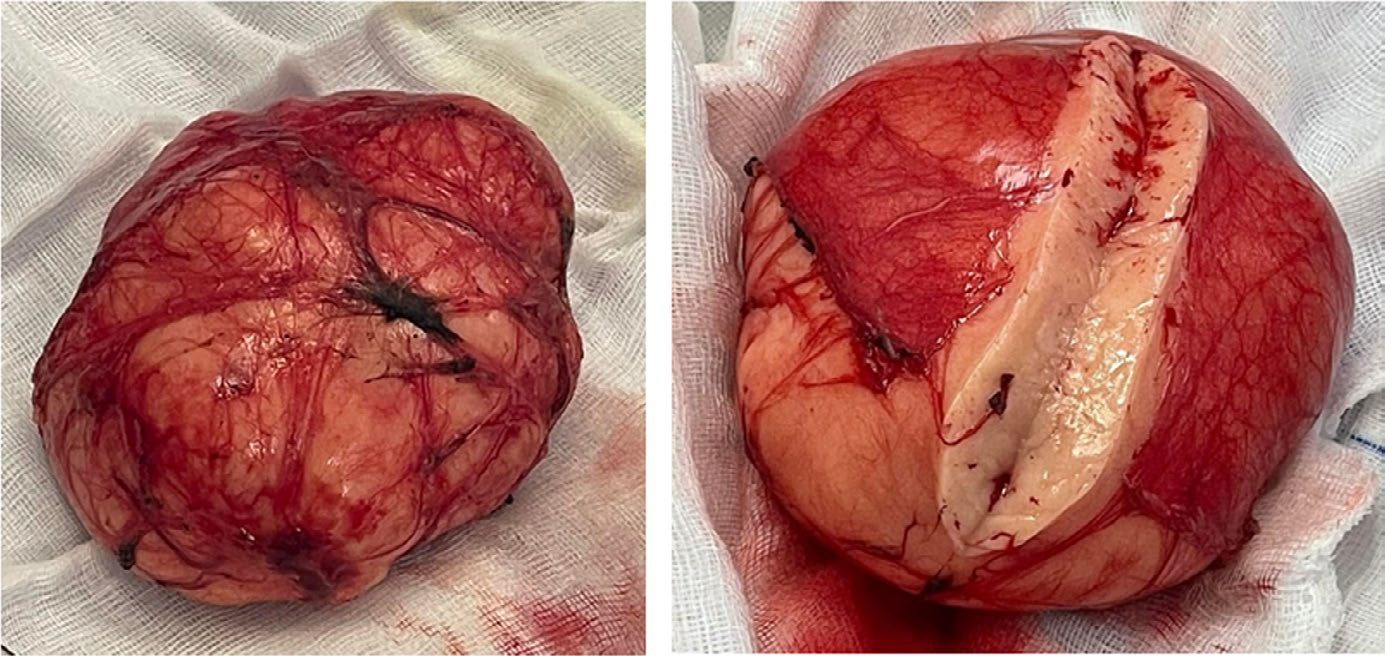
Operative specimen (resected mesenteric lipoma)

Complete excision of the lesion was performed following an avascular plan between the tumor and the ileal mesentery. The mesenteric bed was kept intact with its vascular structures (Figure 3). The pathology of the specimen was consistent with benign lipoma (Figure 4).

The postoperative period was uneventful, and the patient was discharged after three days. Upon follow‐up, the patient was seen regularly for more than 2 years. No recurrence has been diagnosed.

## 3 DISCUSSION

Lipomas are the most common soft tissue tumors of adipocytes. They are commonly located in proximal extremities and the trunk.^1^ Among all small bowel lipomas, mesenteric location accounted for only 4.8% of all cases.^1^

It usually occurs in adults between the fourth and the sixth decade of life^2^ and rarely in children and young people as the case of our patient.^1^, ^3^ There is an increased incidence of lipomas in patients with obesity, diabetes mellitus, hypercholesterolemia, genetic predisposition, and radiation therapy.^3^, ^4^ However, our patient was thin, and none of these factors was found in.

Mesenteric lipomas have generally a slow growth without invasion of surrounding organs.^5^ Besides, due to their soft consistency, most patients are asymptomatic with a chance discovery during abdominal laparotomy or CT scan done for other indications.^1^, ^6^ The onset of symptoms depends on the size, rapidity of growth, and location of the tumor.^1^ An acute abdomen can reveal lipomas as they can lead to intestinal obstruction or volvulus.^1^, ^5^ Less frequently, they can be revealed by chronic abdominal pain as in the case of our patient.

As the first‐line investigation tool for abdominal pain due to its low cost, ultrasound shows homogenous or heterogenous well‐limited and encapsulated intraperitoneal mass, which may be confused with the mesenteric fat.^1^, ^7^ Computed tomography (CT) scan of the abdomen is the gold standard imaging technique and plays a crucial role in the diagnosis of mesenteric lipoma.^1^

It allows the analyses of the nature and the density of the lesion, its exact location, its size, and its extent. It typically shows an intraperitoneal and encapsulated mass with fatty attenuation with no lobulations, septations, or cystic organization.^1^, ^8^ Mesenteric lipomas are often located in the ileal mesentery as in our case.^1^

They must be differentiated from liposarcomas, which have a heterogeneous aspect and contain thick septa with tumor extension to adjacent organs.^9^ Other rare differential diagnosis should be evoked, in particular lipoblastoma, cystic lymphangioma, lymphangiolipoma, and neuroblastoma, but their radiological aspects are different from those of lipomas.^5^

Magnetic resonance imaging (MRI) is also very performant to describe in detail the characteristics of the mass. It shows a hypointensity on T1‐weighted and T2‐weighted images without modification of the signal after injection of gadolinium.^1^ It confirms the fatty nature of the tumor and differentiates giant lipomas from well‐differentiated liposarcomas.^2^ It prevents from resorting to invasive diagnostic techniques such as biopsy before surgery.^2^

Therapeutic management of mesenteric lipoma is not consensual, and it is based on the experience of the different teams. A small lipoma in a asymptomatic patient can be respected.^1^ Symptomatic lipoma with absence of signs of malignancy should be resected with or without the affected intestinal loop to prevent the risk of intestinal obstruction by compression or volvulus.^1^, ^5^ Laparotomy was the most used approach often with resection of the involved bowel followed by end‐to‐end anastomosis.^1^ Laparoscopy may be indicated particularly in cases of small lipoma with a clear plane of division between the tumor and the adjacent small intestine, thus allowing enucleation of the mass.^2^ In our case, CT scan showed a typical benign mesenteric lipoma. Therefore, after an exploratory laparotomy, we performed a complete resection of the mass while conserving the intestine.

The recurrence rate of all lipomas after surgery is less than 5% and is usually due to incomplete excision.^6^

## 4 CONCLUSION

Although mesenteric lipoma is a rare condition reported in the literature, it should be considered in the differential diagnosis of unexplained chronic abdominal pain. Contrast‐enhanced CT scan is the gold standard diagnosis procedure. Treatment is based on complete surgical resection with or without the affected intestinal loop, particularly, in case of symptomatic or large lipoma to prevent the risk of complications.

## 5 SUMMARY

This report describes a rare case of mesenteric lipoma revealed by chronic abdominal pain in 22‐year‐old man. The CT scan images were most consistent with a lipoma and showed no signs of malignancy. Complete excision of the lesion was performed with a clear plan of division between the tumor and the ileal mesentery. Histopathologic examination confirmed the diagnosis of benign lipoma. The postoperative period was uneventful, and no recurrence has been diagnosed. In conclusion, mesenteric lipoma should be considered in the differential diagnosis of unexplained chronic abdominal pain.

## CONFLICT OF INTEREST

None declared.

## AUTHOR CONTRIBUTIONS

Kais Fourati, Amine Zouari, Rami Zouari, and Jawhar Bradai involved in patient management. Aberahmen masmoudi and Jawhar Bradai collected the data. Kais Fourati, Abderahmen Masmoudi, Amine Zouari, and Rami Zouari drafted the manuscript. Najmeddine Hentati and Salah Boujelbene involved in manuscript revision and supervision. All authors read and approved the final manuscript.

## ETHICAL APPROVAL

Personal data have been respected.

## CONSENT

Written informed consent was obtained from the patient to publish this report in accordance with the journal's patient consent policy.

## Data Availability

Personal data of the patient were respected. No data are available for this submission.
